# Supplementary material for: Cell-Penetrating Peptide-Mediated Delivery of Gene-Silencing Nucleic Acids to the Invasive Common Reed Phragmites australis via Foliar Application
Source: Plants (Basel). 2025 Feb 5;14(3):458. doi: 10.3390/plants14030458 (PMC11820330; doi:10.3390/plants14030458)
Supplement: Supplementary file 1 [file plants-14-00458-s001.zip › plants-3329844-supplementary.pdf]

Supplementary Text for

Manuscript:
Cell penetrating peptide-mediated delivery of gene silencing nucleic acids to the invasive common reed Phragmites australis via foliar application

Authors:
Qing Ji[1], Kurt P. Kowalski[2], Edward M. Golenberg[3], Seung Ho Chung[4], Natalie D. Barker[4], Wesley A. Bickford[2], and Ping Gong[4]

Affiliations:
[1] Bennett Aerospace, Inc., Raleigh, NC 27603
[2] U.S. Geological Survey, Great Lakes Science Center, Ann Arbor, MI 48105
[3] Department of Biological Sciences, Wayne State University, Detroit, MI 48201
[4] Environmental Laboratory, U.S. Army Engineer Research and Development Center, Vicksburg, MS 39180

Disclaimer:
Any use of trade, firm, or product names is for descriptive purposes only and does not imply endorsement by the U.S. Government.

---

dsRNA Preparation Protocol

1. Transform pGEM-*PaPDS*, a plasmid expressing *Phragmites australis* phytoene desaturase-coding gene *PaPDS* with a pGEM-T Easy backbone, to *Escherichia coli* (*E. coli*) competent cells

1) Place a dried filter paper disk containing pGEM-*PaPDS*, a plasmid made by cloning a 277-bp *PaPDS* gene fragment to the multiple cloning site (MCS) on a pGEM-T Easy vector (Promega, Madison, WI) (refer to Supplementary Figure 1 for more information), into a 1.5ml tube. Add 0.5 ml TE. Incubate at room temperature for 15 minutes to an hour.
2) Pipette 5 µl of the plasmid to ice thawed competent cells (e.g., JM109 from Promega). Tap the tube briefly. Incubate on ice for 30 minutes.
3) Heat shock at 42°C for exactly 45 seconds. Place back on ice for 2 minutes.
4) Add 500 µl SOC (or LB if SOC is not available). Incubate while shaking horizontally (225 rpm) at 37°C for 1 hour. (We usually tape the tubes to the platform of a shaking incubator.)
5) Spread 100 µl on one LB/AMP plate and the remainder from the tube on a second LB/AMP plate. Incubate at 37°C overnight.
6) Conduct the Blue-White screening next day to select clones containing recombinant DNA (i.e., the pGEM-*PaPDS*). Those non-transformed clones display a blue color, whereas transformed clones display a white color.

2. Plasmid DNA Preparation

1) Grow bacteria in liquid culture. Set up 10.5 ml liquid LB/AMP culture by picking one single white colony from the plate mentioned above and dip in the liquid medium. Incubate at 37°C while shaking at 225 rpm overnight.

Optional: To make a glycerol stock, mix 0.5 ml of overnight bacteria culture with 0.5 ml 50% glycerol solution (25% glycerol in final stock solution). Mix thoroughly and freeze in liquid nitrogen. Store at -80°C.

2) Harvest the cells from the rest of the overnight culture by centrifuge at 4000 rpm for 10 minutes. Discard the supernatant.

3) Use QIAprep Spin Miniprep kit (cat# 27104) (QIAGEN, Germantown, MD) to prepare the plasmid DNA.

4) Check the concentration of plasmid DNA using a NanoDrop spectrophotometry (NanoDrop Technologies, Wilmington, DE).

3. Plasmid DNA verification

1) Digest plasmid DNA with proper restriction enzymes, in this case, using *Sal*I +*Sph*I.

16.0 µl	H_2O (to make a 100 µl final Rx volume)
1.0 µl	Plasmid (200 ng)
2.0 µl	10X reaction buffer
0.5 µl	*Sal*I
0.5 µl	*Sph*I or *Pae*I (an isoschizomer of *Sph*I)
20.0 µl	Total

Incubate the mix at 37°C for 2 hours.

2) Check the digestion products on 1% agarose gel. The DNA fragment insert (~500 bp for *PDS*) should be seen on gel (**Figure S1**).

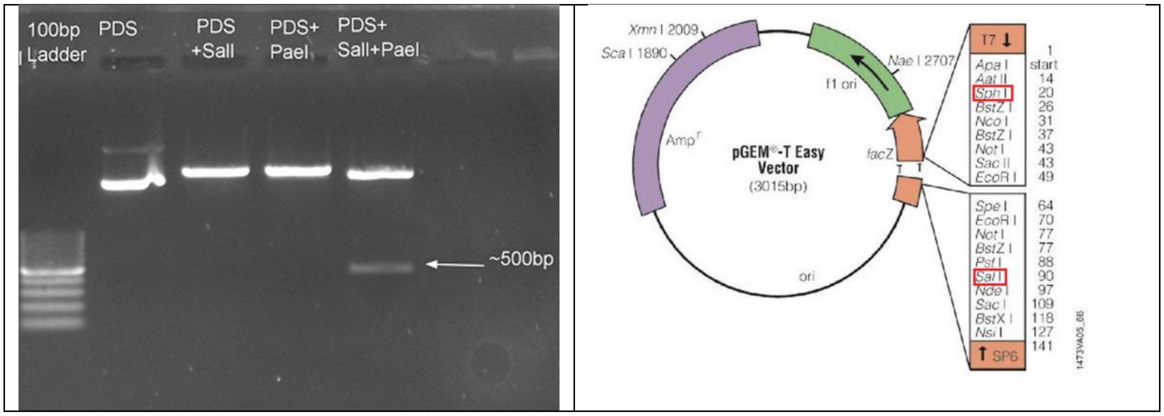

Figure S1. A gel electrophoresis picture showing the restriction enzyme digestion products of the pGEM-*PaPDS* (*Phragmites australis* phytoene desaturase) plasmid DNA (left) and the plasmid's backbone structure of pGEM-T Easy vector (right).

4. Transcription

1) Linearize the plasmid

The pGEM-T Easy plasmid has a T7 promoter on one side of the insertion point and a Sp6 promoter on the other side of the insertion point. For the T7 reaction, prepare a linearized template with *Sal*I. For the Sp6 reaction, prepare a linearized template with *Sph*I.

Digestion reaction

XX µl	H_2O (to make a 100 µl final Rx volume)
YY µl	Plasmid (5 µg)
10.0 µl	10X reaction buffer (specific for either *Sal*I or *Sph*I)
5.0 µl	Restriction enzyme (either *Sal*I or *Sph*I)
100.0 µl	Total

Incubate at 37°C for 2 hours. Kill/deactivate the enzyme by heating at 70°C in a water bath for 10 minutes.

Check the digestion result by running a 1% gel (refer to **Figure S1**). Compare with plasmid before digestion, make sure all plasmid DNA is digested. A small amount of circular plasmid will cause much larger transcription products.

Use DNAclear Purification Kit (cat# AM1756) (Applied Biosystems, Waltham, MA) to clean up the digestion products (linearized plasmids)

2) Transcription

The MAXIscript^{TM} SP6/T7 Transcription Kit (cat# AM1322) (Applied Biosystems) was used for the transcription.

Measure the concentration of the linearized plasmids after cleanup. Try to use 1 µg of template or more per reaction. Set up the digestion reaction as follows:

x µl	*Sal*I digested cleaned-up plasmid (1 µg)
2.5 µl	10X transcription buffer
1 µl	each of 10 mM ATP, GTP, CTP, UTP*
2 µl	T7 RNA polymerase mix
To 25 µl	Water

x µl	*Sph*I digested cleaned-up plasmid (1 µg)
2.5 µl	10X transcription buffer
1 µl	each of 10mM ATP, GTP, CTP, UTP*
2 µl	Sp6 RNA polymerase mix
To 25 µl	Water

Incubate at 37°C for 1 hour.

Add 1µl Turbo DNase and incubate at 37°C for 15 minutes.
Add 1µl 0.5 M EDTA.

* If using labeled UTP, put in the labeled UTP in a 4:6 ratio with unlabeled UTP.

3) Make dsRNA
Combine transcription reactions from both directions. Heat to 70°C in a water bath for 10 minutes and then slowly cool down to room temperature.

4) Cleanup of dsRNA
Use RNeasy mini kit (cat# 74104) (QIAGEN) to purify dsRNA generated in step 3. Measure dsRNA concentration using the NanoDrop.
Set A320 nm as baseline correction, Factor as 50. Calculate the dsRNA concentration and the absorbance ratio using the formulas below:
dsRNA concentration = (A260/A320) x 50 x dilution factor, ng/µl
Absorbance ratio = (A260-A320)/(A280-A320)

An absorbance ratio of ~1.8 is generally accepted as pure for DNA (dsRNA nucleotide). An absorbance ratio below this value indicates the presence of impurity in the sample. For more information, refer to **T042 - TECHNICAL BULLETIN: Nano Drop Spectrophotometers 260/280 and 260/230 Ratios** (https://safe.menlosecurity.com/doc/docview/viewer/docN08607CBD6B94b6681 25e92bf838c9b858d033969e0d18087865eb071a8a47db5a08b0e0aecec)

References:

1) Gurevich VV, Pokrovskaya ID, Obukhova TA, Zozulya SA. 1991. Preparative in vitro mRNA synthesis using SP6 and T7 RNA polymerases. Anal Biochem. 195(2):207-13.
2) Kulkarni MM, Booker M, Silver SJ, Friedman A, Hong P, Perrimon N, Mathey-Prevot B. 2006. Evidence of off-target effects associated with long dsRNAs in *Drosophila melanogaster* cell-based assays. Nat Methods. 3(10):833-8.
3) Roberts-Galbraith RH. 2022. RNAi Screening to Assess Tissue Regeneration in Planarians. In: Blanchoud S, Galliot B, editors. Whole-Body Regeneration: Methods and Protocols [Internet]. New York (NY): Humana; 2022. Fig. 2, [Molecular strategy for dsRNA synthesis...]. doi: 10.1007/978-1-0716-2172-1_27. Available from: https://www.ncbi.nlm.nih.gov/books/NBK586917/figure/ch27.Fig2/

Title Page for Supplementary Figures

- Manuscript Title: Cell penetrating peptide-mediated delivery of gene silencing nucleic acids to the invasive common reed Phragmites australis via foliar application

- Authors: Qing Ji[1], Kurt P. Kowalski[2], Edward M. Golenberg[3], Seung Ho Chung[4], Natalie D. Barker[4], Wesley A. Bickford[2], and Ping Gong[4]
- Affiliation:

  [1] Bennett Aerospace, Inc., Raleigh, NC 27603

  [2] U.S. Geological Survey, Great Lakes Science Center, Ann Arbor, MI 48105

  [3] Department of Biological Sciences, Wayne State University, Detroit, MI 48201

  [4] Environmental Laboratory, U.S. Army Engineer Research and Development Center, Vicksburg, MS 39180

- Disclaimer: Any use of trade, firm, or product names is for descriptive purposes only and does not imply endorsement by the U.S. Government.

>Putative *Phragmites australis* phytoene desaturase (*PaPDS*) gene transcript (788-bp)
AAGCTGGAGAAGTTGGTGGGAGTTCCTGTTATCAATGTTCATATATGGTTTGACAGAAAGCTGAAGAACACATATGACAACCTTCTTTTC
AGCAGGAGTTCACTTTTGAGTGTCTATGCAGACATGTCAGTAACCTGCAAGGAATACTATGATCCAAACCGTTCGATGCTGGAGTTGGTC
TTTGCTCCTGCAGAGGAATGGGTTGGACGAAGTGAAACTGAAATCATCGATGCAACTATGGAAGAGCTAGCAAAATTATTTCCTGACGAA
ATTGCTGCCGATCAAAGTAAAGCTAAGATTCTTAAGTACCATGTTGTGAAGACACCGAGATCTGTTTACAAAACCGTCCCAAATTGTGAA
CCTTGCCGACCTCTCCAAAGATCACCAATTGAAGGCTTCTACCTGGCTGGCGATTACACGAAGCAGAAATACCTGGCTTCCATGGAGGGT
GCAATTCTATCTGGAAAACTTTGCGCCCAGTCTATAGTGCAGGATTATAACATGCTCTCTCTCAGGACCCAGAAAAACCTGCAATCCGAA
ATTCCTGTTGCTTCGTAGTTGTAGTTAGCATCATTCCCACTGGGG[illegible]CTATCATCGCCTGGTCATTTTCTATTTAGTGGGTACCCACCAAC
TACTCACGTAGGAGGAGGACCTTTACAATTCTGTAGAGTTGAATT[illegible]TGACTGAGTTGATATCATATTGGGAAAATGAGATGTAAAACGAC
CTGCATAGCAATTCTTAGACCTTTGCAAAAGGAAAAGTGATAAAG[illegible]GATCGCAGATATTATCTTGCAG

TA cloning (277-bp insert)

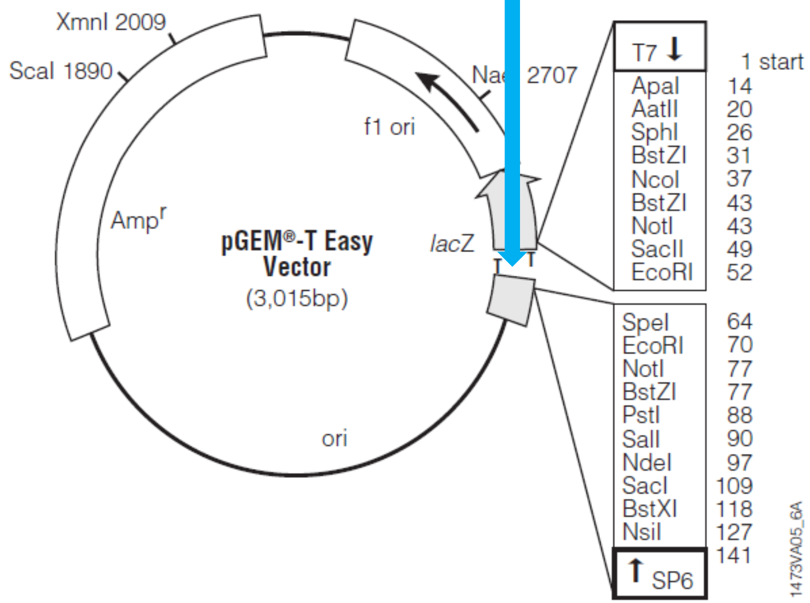

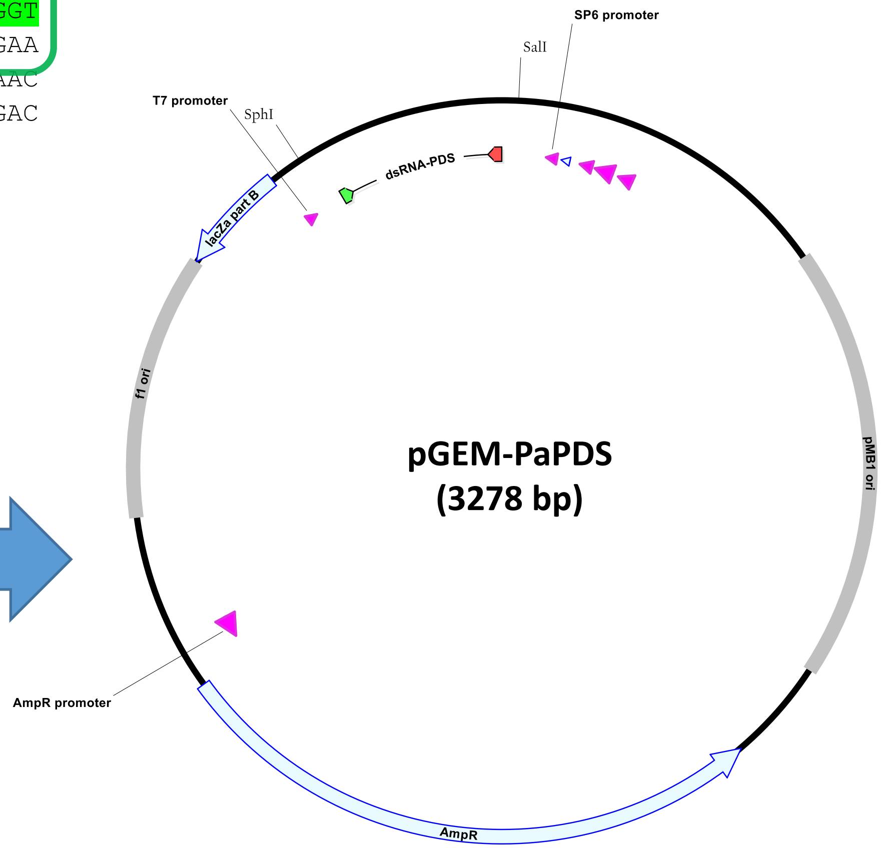

Supplementary Figure S2. Construction of a putative *Phragmites australis* phytoene desaturase (*PaPDS*) gene silencing plasmid pGEM-*PaPDS* by cloning a 277-bp segment of the *PaPDS* gene (green-highlighted) into a pGEM®-T Easy vector.

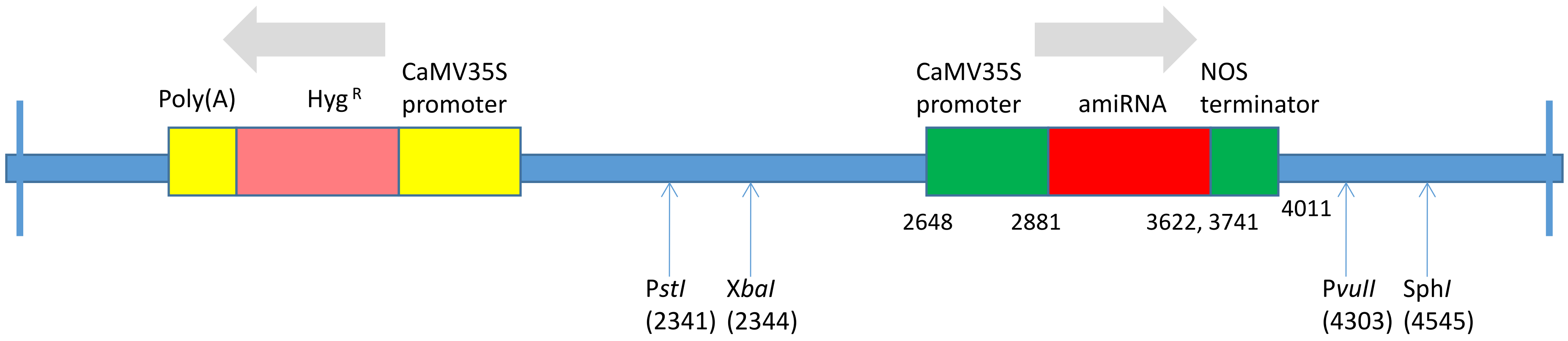

Supplementary Figure S3. Construction map of amiRNA$_{PDS}$ expressing cassettes.
The insert amiRNA$_{PDS1\text{-}5}$ sequences can be found in Table 3.

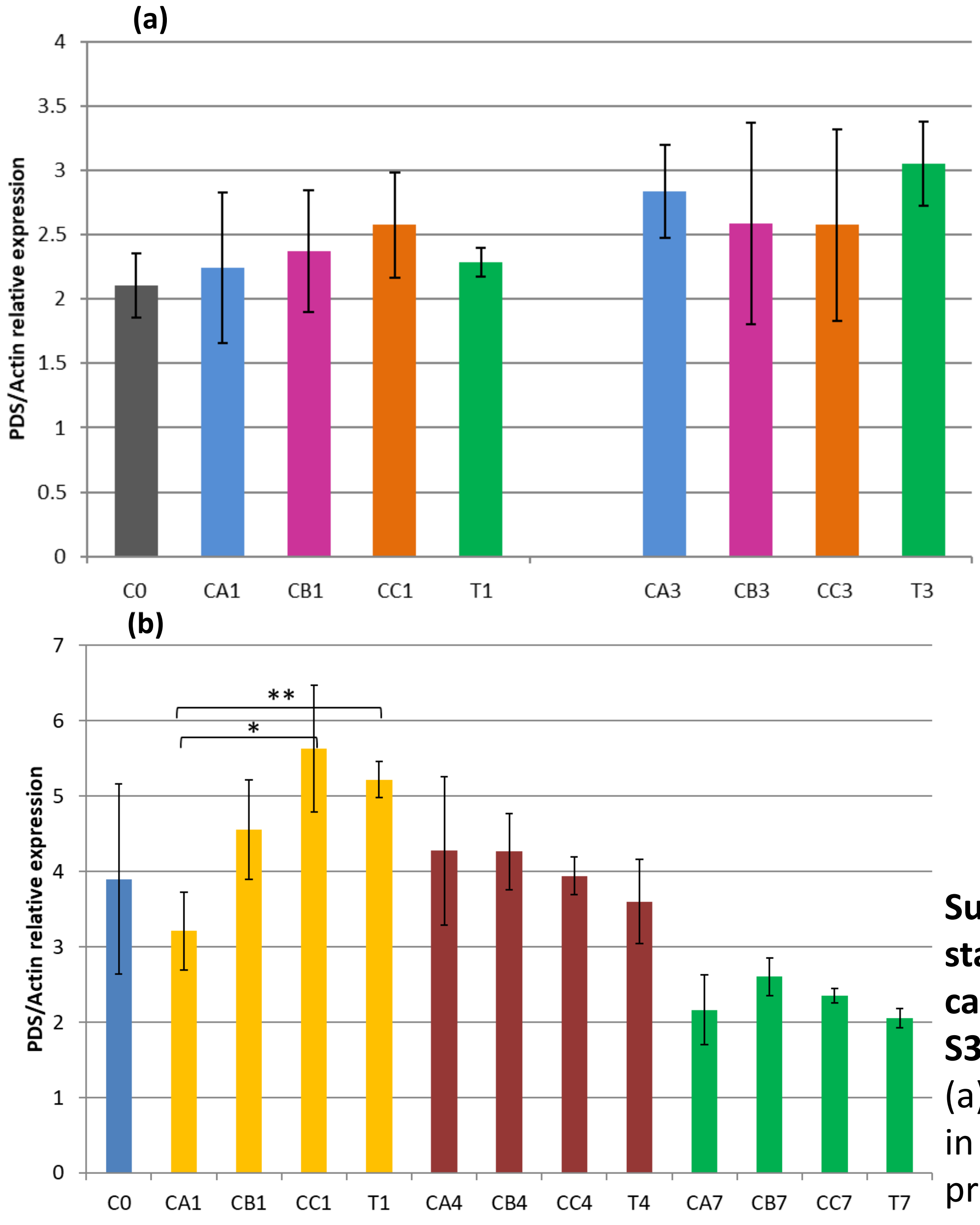

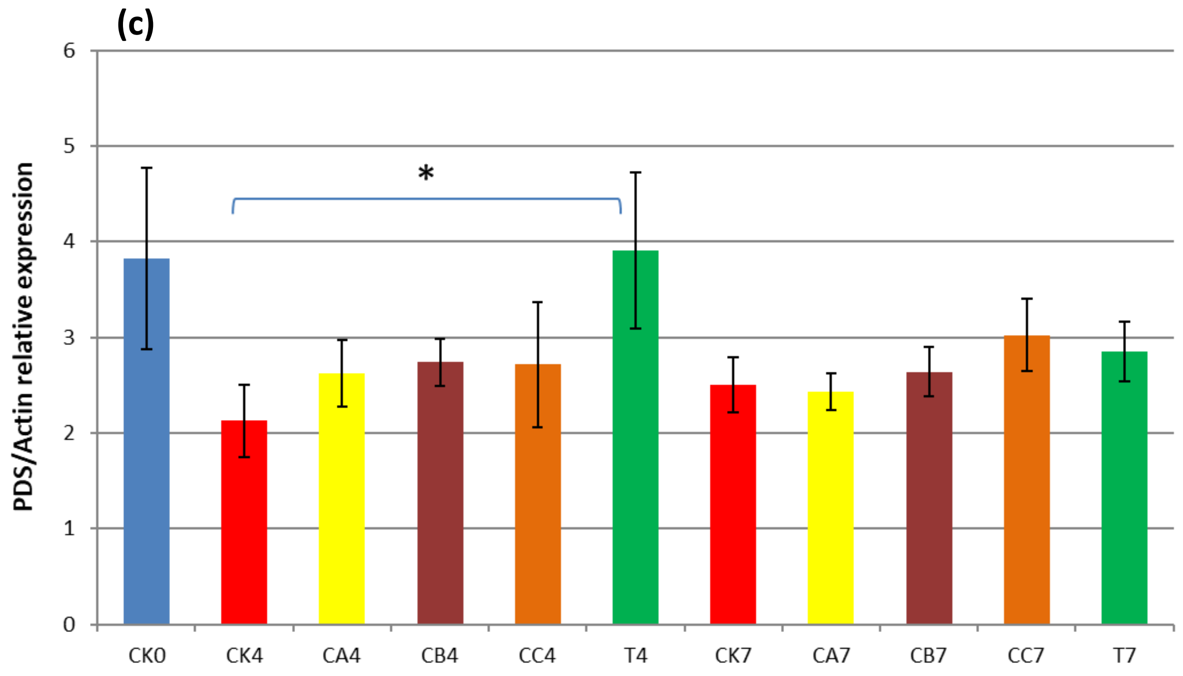

Legend:
C0 or CK0/4/7: Blank control showing the baseline expression level on Day 0/4/7
CA1/3/4/7: Control A with amiRNA and infiltration medium at 1/3/4/7 day(s) post treatment
CB1/3/4/7: Control B with infiltration medium only at 1/3/4/7 day(s) post treatment
CC1/3/4/7: Control C with CPP and infiltration medium at 1/3/4/7 day(s) post treatment
T1/3/4/7: Treatment with amiRNA, CPP and infiltration medium at 1/3/4/7 day(s) post treatment
Error bar: standard deviation; *** and **:** significant difference at $p < 0.05$ and $p < 0.01$, respectively (ANOVA with Tukey post hoc test, n = 3)

Supplementary Figure S4. Relative PDS/Actin expression level (mean ± standard deviation, n = 3) in CPP + amiRNA$_{PDS\text{-}1}$ expressing plasmid (a &b) or cassette (c) treated *Phragmites* leaf samples. Refer to **Supplementary Figure S3** for construct details and **Table 3** for amiRNA$_{PDS\text{-}1}$ sequence. CPP = CADY in (a) and γ-zein-CADY in (b, c). The CPP:GSA charge ratio was 1:1 in (a) and 50:1 in (b, c). The amiRNA$_{PDS\text{-}1}$ expressing cassette only contains CaMV35S promoter, amiRNA insert and NOS terminator.

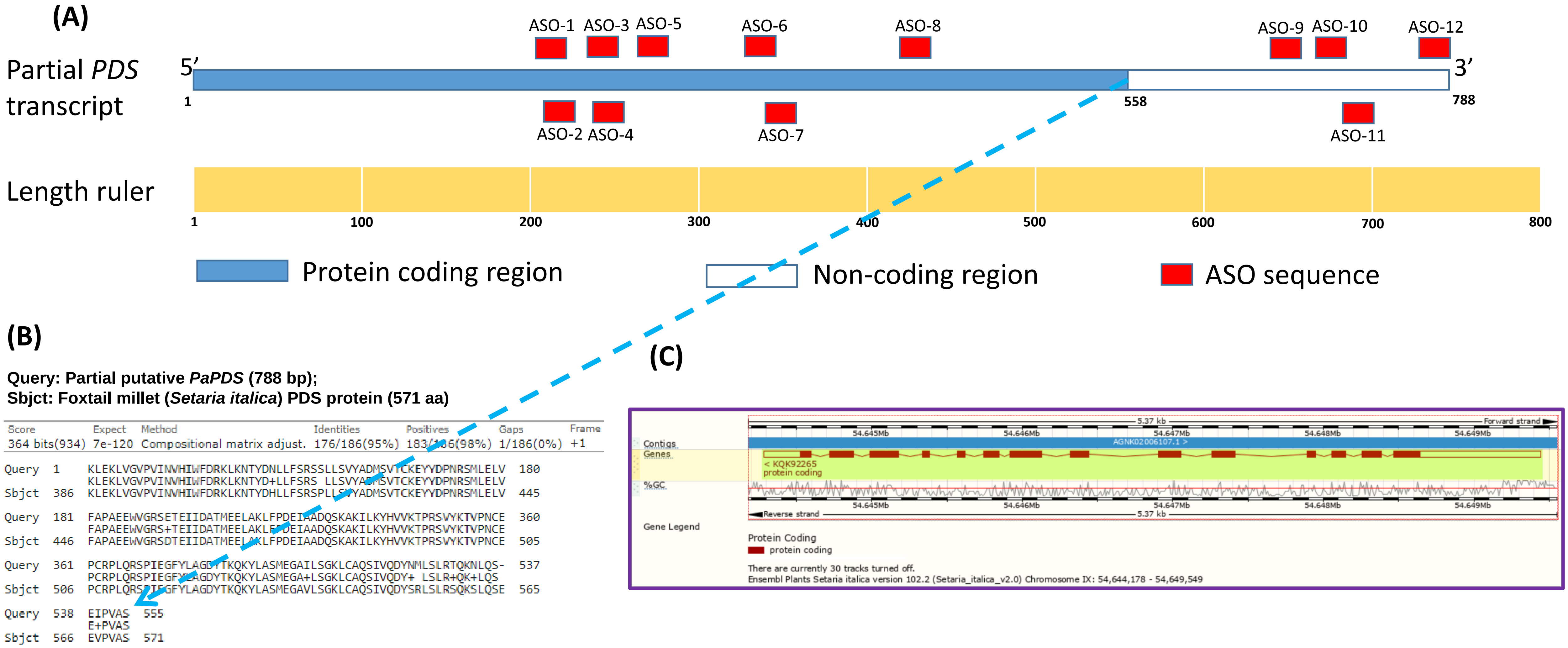

Supplementary Figure S5. (A) Mapping of designed ASOs to the partial putative *P. australis PDS* (*PaPDS*) transcript sequence. (B) Alignment of *PaPDS* against Foxtail millet (*Seraria italica*) PDS protein sequence (BLASTX); (C) Annotated *Seraria italica PDS* gene (SETIT_034914mg) showing the transcript KQK92265 retrieved from the Ensembl Plants database (http://plants.ensembl.org/Setaria_italica/Location/View?db=core;g=SETIT_034914mg;r=IX:54644178-54649549;t=KQK92265)
